# A Potential SARS-CoV-2 Variant of Interest (VOI) Harboring Mutation E484K in the Spike Protein Was Identified within Lineage B.1.1.33 Circulating in Brazil

**DOI:** 10.3390/v13050724

**Published:** 2021-04-21

**Authors:** Paola Cristina Resende, Tiago Gräf, Anna Carolina Dias Paixão, Luciana Appolinario, Renata Serrano Lopes, Ana Carolina da Fonseca Mendonça, Alice Sampaio Barreto da Rocha, Fernando Couto Motta, Lidio Gonçalves Lima Neto, Ricardo Khouri, Camila I. de Oliveira, Pedro Santos-Muccillo, João Felipe Bezerra, Dalane Loudal Florentino Teixeira, Irina Riediger, Maria do Carmo Debur, Rodrigo Ribeiro-Rodrigues, Anderson Brandao Leite, Cliomar Alves do Santos, Tatiana Schäffer Gregianini, Sandra Bianchini Fernandes, André Felipe Leal Bernardes, Andrea Cony Cavalcanti, Fábio Miyajima, Claudio Sachhi, Tirza Mattos, Cristiano Fernandes da Costa, Edson Delatorre, Gabriel L. Wallau, Felipe G. Naveca, Gonzalo Bello, Marilda Mendonça Siqueira

**Affiliations:** 1 Fiocruz COVID-19 Genomic Surveillance Network, Fiocruz, Rio de Janeiro 21040-360, Brazil; paolabmrj@gmail.com (P.C.R.); carolinadiaspaixao@gmail.com (A.C.D.P.); luh.appolinario@gmail.com (L.A.); renata.serranolopes@gmail.com (R.S.L.); anacarolinafmend@gmail.com (A.C.d.F.M.); alicesampaio.br@gmail.com (A.S.B.d.R.); fcm@ioc.fiocruz.br (F.C.M.); ricardo_khouri@hotmail.com (R.K.); camila.indiani@fiocruz.br (C.I.d.O.); pedromuccillo@gmail.com (P.S.-M.); fabio.osv@gmail.com (F.M.); edson.delatorre@ufes.br (E.D.); gabriel.wallau@fiocruz.br (G.L.W.); naveca.felipe@gmail.com (F.G.N.); gbellobr@gmail.com (G.B.); mmsiq@ioc.fiocruz.br (M.M.S); 2 Laboratory of Respiratory Viruses and Measles (LVRS), Oswaldo Cruz Institute, Fiocruz, Rio de Janeiro 21045-900, Brazil; 3 Plataforma de Vigilância Molecular, Instituto Gonçalo Moniz, Fiocruz-BA, Salvador 40296-710, Brazil; 4 Laboratório Central de Saúde Pública do Estado do Maranhão (LACEN-MA), São Luís 65020-904, Brazil; lidio.neto@outlook.com; 5 Laboratório de Enfermidades Infecciosas Transmitidas por Vetores, Instituto Gonçalo Moniz, Fiocruz-BA, Salvador 40296-710, Brazil; 6 Laboratório de Patologia e Biologia Molecular, Instituto Gonçalo Moniz, Fiocruz-BA, Salvador 40296-710, Brazil; 7 Laboratório de Vigilância Molecular Aplicada, Escola Técnica de Saúde, Centro de Ciências da Saúde, Universidade Federal da Paraíba, (UFPB), João Pessoa 58051-900, Brazil; jfb_rn@hotmail.com; 8 Laboratório Central de Saúde Pública do Estado da Paraíba (LACEN-PB), João Pessoa 58013-360, Brazil; dalane.lacenpb@gmail.com; 9 Laboratório Central de Saúde Pública do Estado do Paraná (LACEN-PR), São José dos Pinhais 83060-500, Brazil; irinariediger@sesa.pr.gov.br (I.R.); mariadebur@sesa.pr.gov.br (M.d.C.D.); 10 Laboratório Central de Saúde Pública do Estado do Espírito Santo (LACEN-ES), Vitoria 29050-755, Brazil; ro.ribeiro66@gmail.com; 11 Núcleo de Doenças Infecciosas, Universidade Federal do Espírito Santo, Vitoria 29050-625, Brazil; 12 Laboratório Central de Saúde Pública do Estado do Alagoas (LACEN-AL), Maceió 57036-860, Brazil; anderson.leite@icbs.ufal.br; 13 Laboratório Central de Saúde Pública do Estado do Sergipe (LACEN-SE), Aracaju 49020-380, Brazil; cliomar.santos@fsph.se.gov.br; 14 Laboratório Central de Saúde Pública do Estado do Rio Grande do Sul (LACEN-RS), Porto Alegre 90610-000, Brazil; tatiana-gregianini@saude.rs.gov.br; 15 Laboratório Central de Saúde Pública do Estado de Santa Catarina (LACEN-SC), Florianópolis 88010-002, Brazil; sandrabiafe@gmail.com; 16 Laboratório Central de Saúde Pública do Estado de Minas Gerais (LACEN-MG), Belo Horizonte 30510-010, Brazil; andre.leal@funed.mg.gov.br; 17 Laboratório Central de Saúde Pública do Estado do Rio de Janeiro (LACEN-RJ), Rio de Janeiro 20231-092, Brazil; dg@lacen.fs.rj.gov.br; 18 Oswaldo Cruz Foundation (Fiocruz), Branch Ceará, Eusebio 61760-000, Brazil; 19 Instituto Adolfo Lutz (IAL), São Paulo 01246-000, Brazil; labestrategico@ial.sp.gov.br; 20 Laboratório Central de Saúde Pública do Amazonas, Manaus 69020-040, Brazil; tirza_mattos@hotmail.com; 21 Fundação de Vigilância em Saúde do Amazonas, Manaus 69060-000, Brazil; cristianozoo@yahoo.com.br; 22 Departamento de Biologia, Centro de Ciências Exatas, Naturais e da Saúde, Universidade Federal do Espírito Santo, Alegre 29500-000, Brazil; 23 Departamento de Entomologia e Núcleo de Bioinformática, Instituto Aggeu Magalhães (IAM), FIOCRUZ-PE-Recife 50740-465, Brazil; 24 Laboratório de Ecologia de Doenças Transmissíveis na Amazônia (EDTA), Leônidas e Maria Deane Institute, Fiocruz-AM, Manaus 69057-070, Brazil; 25 Laboratório de AIDS e Imunologia Molecular, Oswaldo Cruz Institute, Fiocruz, Rio de Janeiro 21040-900, Brazil

**Keywords:** SARS-CoV-2, E484K, variant of Interest, genomic epidemiology, Brazil

## Abstract

The severe acute respiratory syndrome coronavirus 2 (SARS-CoV-2) epidemic in Brazil was dominated by two lineages designated as B.1.1.28 and B.1.1.33. The two SARS-CoV-2 variants harboring mutations at the receptor-binding domain of the Spike (S) protein, designated as lineages P.1 and P.2, evolved from lineage B.1.1.28 and are rapidly spreading in Brazil. Lineage P.1 is considered a Variant of Concern (VOC) because of the presence of multiple mutations in the S protein (including K417T, E484K, N501Y), while lineage P.2 only harbors mutation S:E484K and is considered a Variant of Interest (VOI). On the other hand, epidemiologically relevant B.1.1.33 deriving lineages have not been described so far. Here we report the identification of a new SARS-CoV-2 VOI within lineage B.1.1.33 that also harbors mutation S:E484K and was detected in Brazil between November 2020 and February 2021. This VOI displayed four non-synonymous lineage-defining mutations (NSP3:A1711V, NSP6:F36L, S:E484K, and NS7b:E33A) and was designated as lineage N.9. The VOI N.9 probably emerged in August 2020 and has spread across different Brazilian states from the Southeast, South, North, and Northeast regions.

## 1. Introduction

The SARS-CoV-2 epidemic in Brazil was mainly driven by lineages B.1.1.28 and B.1.1.33 that probably emerged in February 2020 and were the most prevalent variants in most country regions until October 2020 [1,2]. Recent genomic studies, however, bring attention to the emergence of new SARS-CoV-2 variants in Brazil harboring mutations at the receptor-binding site (RBD) of the Spike (S) protein that might impact viral fitness and transmissibility.

So far, one variant of concern (VOC), designated as lineage P.1, and one variant of interest (VOI), designated as lineage P.2, have been identified in Brazil and both evolved from lineage B.1.1.28. The VOC P.1, first described in January 2021 [3], displayed an unusual number of lineage-defining mutations in the S protein (L18F, T20N, P26S, D138Y, R190S, K417T, E484K, N501Y, H655Y, T1027I) and its emergence was associated with a second COVID-19 epidemic wave in the Amazonas state [4,5]. The VOI P.2, first described in samples from October 2020 in the state of Rio de Janeiro, was distinguished by the presence of the S:E484K mutation in RBD and other four lineage-defining mutations outside the S protein [6]. The P.2 lineage has been detected as the most prevalent variant in several states across the country in late 2020 and early 2021 (https://www.genomahcov.fiocruz.br, accessed on 1 March 2021).

Several B.1.1.33-derived lineages are currently defined by the Pangolin system including: lineage N.1 detected in the US, lineage N.2 detected in Suriname and France, lineage N.3 circulating in Argentina, and lineages N.4 and B.1.1.314 circulating in Chile (https://cov-lineages.org/lineages.html, accessed on 1 March 2021). However, none of these B.1.1.33-derived lineages were characterized by mutations of concern in the S protein. Here, we define the lineage N.9 within B.1.1.33 diversity that harbors mutation E484K in the S protein as was detected in different Brazilian states between November 2020 and February 2021.

## 2. Materials and Methods

The Fiocruz COVID-19 Genomic Surveillance Network has recovered SARS-CoV-2 lineage B.1.1.33 genomes from 422 positive samples between 12th March 2020 and 27th January 2021 (Supplementary Material). Sequencing protocols were as previously described [7,8]. The FASTQ reads obtained were imported into the CLC Genomics Workbench version 20.0.4 (Qiagen A/S, Denmark), trimmed, and mapped against the reference sequence EPI_ISL_402124 available in EpiCoV database in the GISAID (https://www.gisaid.org/, accessed on 1 March 2021). The alignment was refined using the InDels and Structural Variants module. 

Sequences were then combined with 816 B.1.1.33 Brazilian genomes available in the EpiCoV database in GISAID by 1st March 2021 (Supplementary Table S1). Only high quality (<1% of N) complete (>29 kb) SARS-CoV-2 genomes were used. This dataset was then aligned using MAFFT v7.475 [9] and subjected to maximum likelihood (ML) phylogenetic analysis using IQ-TREE v2.1.2 [10] under the GTR + F + G4 nucleotide substitution model, as selected by the ModelFinder application [11]. Branch support was assessed by the approximate likelihood-ratio test based on the Shimodaira–Hasegawa procedure (SH-aLRT) with 1000 replicates. The mutational profile was investigated using the Nextclade tool (https://clades.nextstrain.org, accessed on 1 March 2021) and temporal signal was assessed by the regression analysis of the root-to-tip genetic distance against sampling dates using the program Tempest [12].

A time-scaled phylogenetic tree was estimated using the Bayesian Markov Chain Monte Carlo (MCMC) approach implemented in BEAST 1.10.4 [13]. Bayesian tree was reconstructed using the GTR + F + I + G4 nucleotide substitution model, the non-parametric Bayesian skyline (BSKL) model as the coalescent tree prior and a strict molecular clock model with a uniform substitution rate prior (8 × 10^–4^–10 × 10^–4^ substitutions/site/year). Ancestral node states were reconstructed using a reversible discrete phylogeographic model [14] where transitions between sampling locations (Brazilian states) were estimated in a continuous-time Markov chain (CTMC) rate reference prior. Convergence (effective sample size > 200) in parameter estimates was assessed using TRACER v1.7 18. The maximum clade credibility (MCC) tree was summarized with TreeAnnotator v1.10.4. ML and MCC trees were visualized using FigTree v1.4.4 (http://tree.bio.ed.ac.uk/software/figtree/, accessed on 1 March 2021).

## 3. Results and Discussion

**Figure 1:**
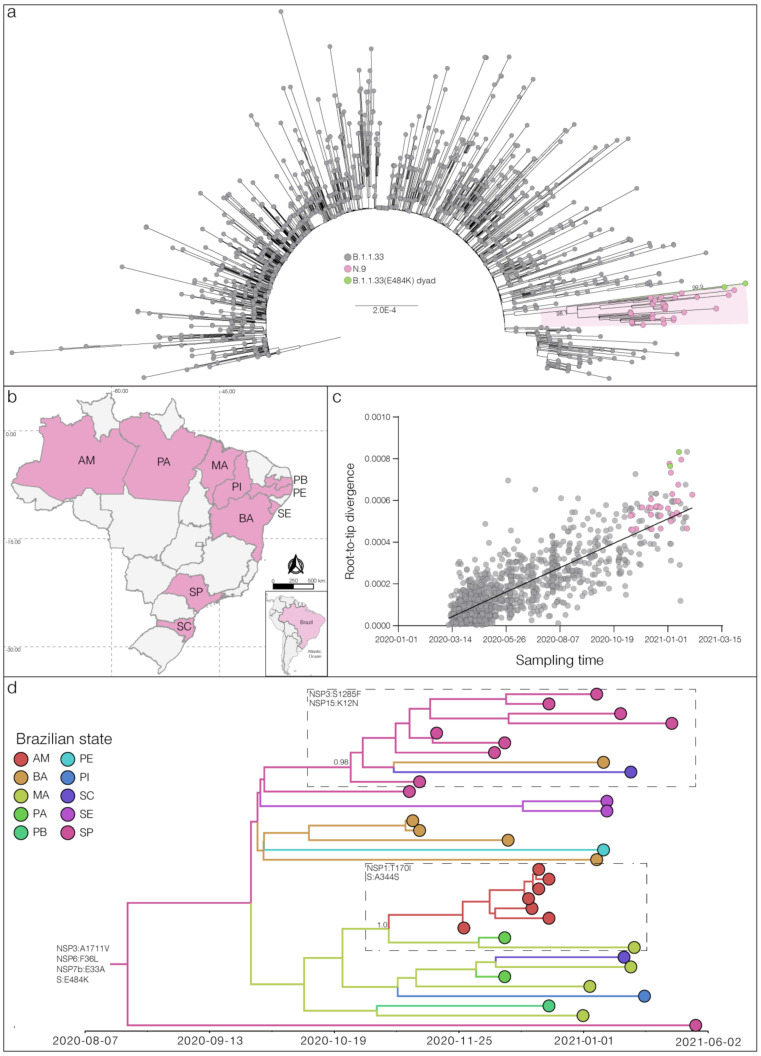
Lineage N.9 evolutionary origin and spatial-temporal distribution. (**a**) Maximum likelihood (ML) phylogenetic tree of the B.1.1.33 whole-genome sequences from Brazil. The B.1.1.33 sequences with mutation S:E484K are represented by pink (VOI N.9) and green (B.1.1.33(E484K)) circles. The SH-aLRT support values are indicated in key nodes and branch lengths are drawn to scale with the left bar indicating nucleotide substitutions per site. (**b**) Geographic distribution of the VOI N.9 identified in Brazil. Brazilian states’ names follow the International Organization for Standardization (ISO) 3166-2 standard. (**c**) Correlation between the sampling date of B.1.1.33 sequences and their genetic distance from the root of the ML phylogenetic tree. Colors indicate the B.1.1.33 clade as indicated in (**a**). (**d**) Bayesian phylogeographic analysis of N.9 lineage. Tips and branches colors indicate the sampling state and the most probable inferred location of their descendent nodes, respectively, as indicated in the legend. Branch posterior probabilities are indicated in key nodes. Boxes highlight two N.9 subclades carrying additional mutations (indicated in each box). The tree was automatically rooted under the assumption of a strict molecular clock, and all horizontal branch lengths are time-scaled.

Mutation profile analysis revealed a total of 34 B.1.1.33 sequences harboring the S:E484K mutation. ML phylogenetic analysis revealed that 32 of these sequences branched in a highly supported (SH-aLRT = 98%) monophyletic clade that define a potential new VOI designated as N.9 PANGO lineage [15]. The other two sequences harboring the S:E484K mutation branched separately in a highly supported (SH-aLRT = 100%) dyad (Figure 1a). The VOI N.9 is characterized by four non-synonymous lineage-defining mutations (NSP3:A1711V, NSP6:F36L, S:E484K, and NSP7b:E33A) and also contains a group of three B.1.1.33 sequences from the Amazonas state that has no sequencing coverage in the position 484 of the S protein, but share the remaining N.9 lineage-defining mutations (Table 1), thus forming a cluster of 35 sequences. The B.1.1.33 (S:E484K) dyad comprises two sequences from the Maranhao state and were characterized by a different set of non-synonymous mutations (Supplementary Table S2).

Among the 35 genomes identified so far as VOI N.9, 10 Brazilian states were represented, suggesting that this lineage is already highly dispersed in the country. The VOI N.9 was first detected in Sao Paulo state on 11 November 2020, and soon later in other Brazilian states from the South (Santa Catarina), North (Amazonas and Para), and Northeast (Bahia, Maranhao, Paraiba, Pernambuco, Piaui, and Sergipe) regions (Figure 1b). Analysis of the temporal structure revealed that the overall divergence of lineage N.9 is consistent with the substitution pattern of other B.1.1.33 sequences (Figure 1c), thus suggesting no unusual accumulation of mutations in this VOI. Molecular clock analysis estimated the emergence of the VOI N.9 most probably in the states of Sao Paulo (Posterior State Probability (PSP) = 0.42), Bahia (PSP = 0.32) or Maranhao (PSP = 0.18) at 15th August, 2020 (95% High Posterior Density (HPD): 16th June–22th September, 2020) (Figure 1d). This analysis also revealed that some additional mutations were acquired during evolution of VOI N.9 in Brazil, determining two highly supported (PP > 0.95) subclades. One subclade, that mostly contains sequences from Sao Paulo state, probably arose on 16th October (95% HPD: 22th September–5th November) and was defined by additional mutations NSP3:S1285F and NSP15:K12N. The other subclade that mostly comprises sequences from the North region probably arose on 29th October (95% HPD: 5th October–17th November) and was defined by additional mutations NSP1:T170I and S:A344S (Figure 1d).

## 4. Conclusions

In this study we identified the emergence of a new VOI (S:E484K) within lineage B.1.1.33 circulating in Brazil. The VOI N.9 displayed a low prevalence (~3%) among all Brazilian SARS-CoV-2 samples analyzed between November 2020 and February 2021, but it is already widely dispersed in the country and comprises a high fraction (35%) of the B.1.1.33 sequences detected in that period. Mutation S:E484K has been identified as one of the most important substitutions that could contribute to immune evasion as confers resistance to several monoclonal antibodies and also reduces the neutralization potency of some polyclonal sera from convalescent and vaccinated individuals [16,17,18]. Mutation S:E484K has emerged independently in multiple VOCs (P.1, B.1.351 and B.1.1.7) and VOIs (P.2 and B.1.526) [19] spreading around the world, and it is probably an example of convergent evolution and ongoing adaptation of the virus to the human host.

The onset date of VOI N.9 here estimated around mid-August roughly coincides with the estimated timing of emergence of the VOI P.2 in late-July 6 and shortly precede the detection of a major global shift in the SARS-CoV-2 fitness landscape after October 2020 [20]. These findings indicate that 484K variants probably arose simultaneously in the two most prevalent viral lineages circulating in Brazil around July–August, but may have only acquired some fitness advantages, which accelerated its dissemination, after October 2020. We predict that the Brazilian COVID-19 epidemic during 2021 will be dominated by a complex array of B.1.1.28 (S:E484K), including P.1 and P.2, and B.1.1.33 (S:E484K) variants that will completely replace the parental 484E lineages that drove the epidemic in 2020. Implementation of efficient mitigation measures in Brazil is crucial to reduce community transmission and prevent the recurrent emergence of more transmissible variants that could further exacerbate the epidemic in the country.

## Figures and Tables

**Table 1:** Synapomorphic mutations of SARS-CoV-2 lineage N.9.

Genomic Region (Protein)	Nucleotide	Amino Acid
ORF1a	G1264T	-
ORF1a	C7600T	-
ORF1a (NSP3)	C7851T	A2529V (A1711V)
ORF1a (NSP6)	T11078C	F3605L (F36L)
Spike (S)	G23012A	E484K
ORF7b (NSP7b)	A27853C	E33A

## Data Availability

All genomes generated in this work were deposited in the EpiCoV database of GISAID (https://www.gisaid.org/, accessed on 1 March 2021). Accession codes are available in Supplementary Material.

## Supplementary Materials

The following are available online at https://www.mdpi.com/article/10.3390/v13050724/s1, Supplementary Material: GISAID accession numbers of genomes lineage B.1.1.33 from this study, Supplementary Table S1: GISAID Acknowledgement Table, Supplementary Table S2: Synapomorphic non-synonymous mutations of the B.1.1.33(S:E484K) dyad isolated in the state of Maranhao.
