# Supplementary material for: A Potential SARS-CoV-2 Variant of Interest (VOI) Harboring Mutation E484K in the Spike Protein Was Identified within Lineage B.1.1.33 Circulating in Brazil
Source: Viruses. 2021 Apr 21;13(5):724. doi: 10.3390/v13050724 (PMC8143327; doi:10.3390/v13050724)
Supplement: Supplementary file 1 [file viruses-13-00724-s001.zip › Resende_20210317_SupMaterial/SupplementaryMaterial.docx]

**Supplementary Material.** GISAID accession numbers of genomes lineage B.1.1.33 from this study:

EPI_ISL_427294 to EPI_ISL_427298; EPI_ISL_427302 to EPI_ISL_427304; EPI_ISL_456071 to EPI_ISL_456077; EPI_ISL_456079 to EPI_ISL_456082; EPI_ISL_456084 to EPI_ISL_456087; EPI_ISL_456089 to EPI_ISL_456096; EPI_ISL_456098 to EPI_ISL_456106; EPI_ISL_467345; EPI_ISL_467347 to EPI_ISL_467353; EPI_ISL_467355; EPI_ISL_467357; EPI_ISL_467358; EPI_ISL_467360 to EPI_ISL_467365; EPI_ISL_467367 to EPI_ISL_467371; EPI_ISL_541347 to EPI_ISL_541350; EPI_ISL_541352; EPI_ISL_541353; EPI_ISL_541356 to EPI_ISL_541358; EPI_ISL_541360 to EPI_ISL_541365; EPI_ISL_541367; EPI_ISL_541369; EPI_ISL_541370; EPI_ISL_541376; EPI_ISL_541382; EPI_ISL_541385; EPI_ISL_541388; EPI_ISL_541392; EPI_ISL_541397 to EPI_ISL_541399; EPI_ISL_729794 to EPI_ISL_729798; EPI_ISL_729800; EPI_ISL_729802; EPI_ISL_729807; EPI_ISL_729809; EPI_ISL_729810; EPI_ISL_729812; EPI_ISL_729814; EPI_ISL_729816; EPI_ISL_729817 to EPI_ISL_729821; EPI_ISL_729823; EPI_ISL_729825; EPI_ISL_729826 to EPI_ISL_729833; EPI_ISL_729837 to EPI_ISL_729839; EPI_ISL_729841 to EPI_ISL_729844; EPI_ISL_729849; EPI_ISL_729851; EPI_ISL_729857; EPI_ISL_729858; EPI_ISL_729860; EPI_ISL_792561; EPI_ISL_792571 to EPI_ISL_792573; EPI_ISL_792579; EPI_ISL_792583; EPI_ISL_792588; EPI_ISL_792589; EPI_ISL_792593; EPI_ISL_792594; EPI_ISL_792596; EPI_ISL_792600; EPI_ISL_792601 to EPI_ISL_792603; EPI_ISL_792608; EPI_ISL_792610; EPI_ISL_792612; EPI_ISL_792623; EPI_ISL_792637; EPI_ISL_792640; EPI_ISL_792644; EPI_ISL_792648; EPI_ISL_1181353 to EPI_ISL_1181624.
