# Supplementary material for: A Potential SARS-CoV-2 Variant of Interest (VOI) Harboring Mutation E484K in the Spike Protein Was Identified within Lineage B.1.1.33 Circulating in Brazil
Source: Viruses. 2021 Apr 21;13(5):724. doi: 10.3390/v13050724 (PMC8143327; doi:10.3390/v13050724)
Supplement: Supplementary file 1 [file viruses-13-00724-s001.zip › Resende_20210317_SupMaterial/SupplementaryTableS2.docx]

| Genomic region | Amino Acid | Deletions |
| --- | --- | --- |
| ORF1a (NSP3) | P1640L (P822L) |  |
| ORF1a (NSP5) | P3371S (P108S) |  |
| ORF1a (NSP5) | P3395S (P132S) |  |
| ORF1a (NSP6) | V3718A (V149A) |  |
| Spike | P9L |  |
| Spike |  | 141-144 |
| Spike | I210V |  |
| Spike |  | 211 |
| Spike | L212I |  |
| Spike |  | 256-258 |
| Spike | V445A |  |
| Spike | E484K |  |
| ORF7b |  | 14 |
| N | Q70R |  |

**Supplementary Table S2.** Synapomorphic non-synonymous mutations of the B.1.1.33(S:E484K) dyad isolated in the state of Maranhao.
